# Identifying the ‘active ingredients’ of socioeconomic disadvantage for youth outcomes in middle childhood

**DOI:** 10.1017/S0954579423000135

**Published:** 2023-02-27

**Authors:** Sarah L. Carroll, Elizabeth A. Shewark, Megan E. Mikhail, Daniel J. Thaler, Amber L. Pearson, Kelly L. Klump, S. Alexandra Burt

**Affiliations:** 1 Department of Psychology, Michigan State University, East Lansing, MI, USA; 2 Department of Geography, Environment and Spatial Sciences, Michigan State University, East Lansing, MI, USA

**Keywords:** academic achievement, ecological model, neighborhood context, socioeconomic disadvantage, youth psychopathology

## Abstract

**Background:**

Youth experiencing socioeconomic deprivation may be exposed to disadvantage in multiple contexts (e.g., neighborhood, family, and school). To date, however, we know little about the underlying structure of socioeconomic disadvantage, including whether the 'active ingredients' driving its robust effects are specific to one context (e.g., neighborhood) or whether the various contexts increment one another as predictors of youth outcomes.

**Methods:**

The present study addressed this gap by examining the underlying structure of socioeconomic disadvantage across neighborhoods, families, and schools, as well as whether the various forms of disadvantage jointly predicted youth psychopathology and cognitive performance. Participants were 1,030 school-aged twin pairs from a subsample of the Michigan State University Twin Registry enriched for neighborhood disadvantage.

**Results:**

Two correlated factors underlay the indicators of disadvantage. Proximal disadvantage comprised familial indicators, whereas contextual disadvantage represented deprivation in the broader school and neighborhood contexts. Results from exhaustive modeling analyses indicated that proximal and contextual disadvantage incremented one another as predictors of childhood externalizing problems, disordered eating, and reading difficulties, but not internalizing symptoms.

**Conclusions:**

Disadvantage within the family and disadvantage in the broader context, respectively, appear to represent distinct constructs with additive influence, carrying unique implications for multiple behavioral outcomes during middle childhood.

## Introduction

Although often conceptualized as a unitary construct (e.g., Brooks-Gunn, 1997), socioeconomic disadvantage may manifest at multiple levels, including neighborhood (e.g., % of households in a given Census-tract living in poverty), family (e.g., low household income), and school (e.g., high subsidized lunch rate). Each form of disadvantage has been independently shown to predict youth psychopathology and academic outcomes (e.g., Brooks-Gunn, 1997; Christle et al., 2005; Kupersmidt et al., 1995; Rivenbark et al., 2019). For example, children residing in impoverished neighborhoods typically exhibit higher rates of psychopathology and lower academic achievement relative to their peers in wealthier neighborhoods (Gordon & Cui, 2018; Kupersmidt et al., 1995; Xue et al., 2005). Likewise, children from impoverished families demonstrate lower academic achievement than their peers (Hair et al., 2015) and higher rates of psychopathology, particularly externalizing (Peverill et al., 2021). Very few studies have examined school-level disadvantage, but there is preliminary evidence linking school characteristics (e.g., subsidized lunch rate) to youth behavior problems (Christle et al., 2005) and academic performance (Caldas & Bankston, 1997).

In part because of the consistency of these associations, the (generally implicit) assumption has been that the various forms of disadvantage serve as different indicators of the same phenomenon – poverty. This assumption requires empirical testing, however, as it may not accurately describe the phenomenology of socioeconomic disadvantage. Associations among neighborhood, family, and school disadvantage, for example, are typically low-to-moderate (*rs*. .20–.40; Mode et al., 2016), such that not all families residing in a disadvantaged neighborhood are themselves impoverished (or vice versa). There is also some preliminary evidence that the various forms of disadvantage differentially predict behavioral outcomes. For example, prior research has found that neighborhood poverty (Kupersmidt et al., 1995) and family instability (Ackerman et al., 1999) predict child antisocial behavior even after accounting for familial poverty. Furthermore, the impact of school-level poverty (e.g., percentage of students eligible for subsidized lunch) on standardized test scores persists after accounting for familial poverty and parental education and occupation (Caldas & Bankston, 1997). These limited empirical findings collectively suggest that the various forms of socioeconomic disadvantage may not be interchangeable.

The relative absence of research in this area is made all the more surprising by the fact that such findings are consistent with Bronfenbrenner’s ecological theory, which posits that proximal and distal contexts independently impact development (Bronfenbrenner, 1988). Within Bronfenbrenner’s framework, individual development is embedded in multiple contexts. The microsystem, or immediate context, comprises the family and school community, whereas the neighborhood is part of the exosystem or broader social context. The individual interacts differently within each context, and the contexts themselves may interact when shaping development. In short, prior theoretical work suggests that the processes conducive to the development of psychopathology and academic difficulties may differ across neighborhoods, homes, and schools.

Although the above empirical and theoretical work supports the presence of meaningful differences within the broader construct of disadvantage, far more work is needed. The studies discussed above either examined multiple indices of disadvantage within a single domain (e.g., familial disadvantage; Ackerman et al., 1999) or restricted their analyses to youth antisocial behavior outcomes (Kupersmidt et al., 1995). Moreover, extant research incorporating school indices of disadvantage is limited. Although school-level poverty was found to predict cognitive performance above the effects of familial disadvantage (Caldas & Bankston, 1997), the simultaneous role of neighborhood remains unexplored. Such work would be especially important to do in states like Michigan that allow school of choice (i.e., children can enroll in public schools outside of their own district), since neighborhood residence and school deprivation may not overlap.

In sum, prior studies have not evaluated whether neighborhood, family, and school disadvantage constitute separable domains of disadvantage, nor have they examined disadvantage as a multifaceted predictor of multiple youth outcomes. As such, it is unclear whether the various dimensions of disadvantage act as joint or independent predictors of youth outcomes. For example, it is possible that disadvantage in schools and neighborhoods might overlap as predictors of antisocial behavior, and do so above and beyond familial disadvantage. Likewise, given that the effects of school-level disadvantage on academic achievement persisted when controlling for familial disadvantage (Caldas & Bankston, 1997), youth residing in disadvantaged homes and neighborhoods and attending impoverished schools may be at elevated risk for academic difficulties relative to their neighbors attending better-resourced schools in another district. Findings clarifying *which* aspects of disadvantage have the strongest effects on youth outcomes could have important implications for policy and intervention.

### Present study

The present study sought to address these gaps in the literature. We first sought to clarify the underlying structure of disadvantage, examining familial and neighbor-informant reports of disadvantage, Census block-group data and school administrative data in a sample of 1,030 school-aged twin pairs enriched for neighborhood disadvantage. We then examined the various forms of disadvantage as joint predictors of externalizing behaviors, internalizing symptoms, disordered eating, and cognitive performance. Given the number of predictors and outcomes, we conducted our analyses using an exhaustive modeling approach that examines all available data specifications (i.e., type of disadvantage, youth outcome, and control for demographic confounds) (Simonsohn et al., 2020). Given the robust effects of neighborhood, school, and familial disadvantage, respectively, on youth outcomes, we hypothesized that each broad form of disadvantage would represent a unique construct and would contribute uniquely to each behavioral outcome under study.

## Method

### Participants

Participants were drawn from the Twin Study of Behavioral and Emotional Development in Children (TBED-C), a study within the population-based Michigan State University Twin Registry (MSUTR) (Burt & Klump, 2019). The TBED-C includes both a population-based subsample (*N* = 528 families) and an independent at-risk subsample for which inclusion criteria specified that participating twin families lived in neighborhoods with neighborhood poverty levels at or above the Census mean at study onset (10.5%) (*N* = 502 families). To be eligible for participation, neither twin could have a cognitive or physical condition (as assessed via parental screen; e.g., significant developmental delay) that would preclude completion of the assessment. Other recruitment details are reported at length in prior publications (e.g., Burt & Klump, 2019). Most assessments were conducted between 2008 and 2012.

Participants ranged in age from 6 to 11 years (mean = 8.06, SD = 1.45) and were 48.7% female. Families identified as White: 81.7%, Black: 9.5%, Asian/Pacific Islander: 1.1%, Hispanic: 0.7%, Native American: 1.1%, and multiracial: 5.9%. These proportions are largely consistent with those for the population of the State of Michigan (http://www.Census.gov/) (e.g., White: 79%, Black: 14%). Notably, Michigan implemented a “school of choice” policy beginning in 1994, meaning that participants residing in disadvantaged neighborhoods may not necessarily attend their local schools (Plank & Sykes, 1999).

### Ethical considerations

The TBED-C was approved by the Michigan State University IRB. Children provided informed assent and parents provided informed consent for themselves and their children.

## Measures

### Disadvantage

#### Neighborhood disadvantage.

Neighborhood disadvantage was assessed in three ways: The Area Deprivation Index (ADI), neighbor-informant reports of neighborhood problems, and participating mothers’ reports of neighborhood problems. The ADI (Kind & Buckingham, 2018) comprises 17 measures of neighborhood disadvantage (see Table S1) at the block-group level using data from the 2008 to 2012 American Community Survey of the US Census. Data were weighted according to the factor loadings identified by Kind & Buckingham (2018), and the weighted variables were summed to create a deprivation index score for each block group. Participating families were assigned a percentile score indicating the level of deprivation in their block group relative to that of all US block groups.

We also recruited 10 randomly chosen neighbors from each at-risk family’s Census tract to complete a survey regarding their perceptions of their community. Neighbors completed the 13-item Extent of Neighborhood Problems scale (α = .95), reporting whether issues such as graffiti and violent crime were problems in their community using a 5-point Likert scale (1 = strongly agree to 5 = strongly disagree) (Henry et al., 2014). Responses were reverse-coded so that higher scores indicated greater disadvantage. We then geocoded and mapped neighbor and twin family addresses using ArcGIS v10.3 (ESRI, 2020). Average perceptions of neighborhood disadvantage, based on reports from neighbors residing within a 5 km radius of participating families, were calculated for each family (in both subsamples). The mean number of neighbors living within 5 km of a participating family was 13.09 (SD = 10.98), with a median of 10 (range: 1–47). Lastly, participating mothers in both subsamples also completed the Extent of Neighborhood Problems scale (α = .95).

#### School disadvantage.

School disadvantage was assessed via two indices: subsidized lunch rate and average performance on standardized tests (neither of which were among the indicators in the ADI). Parents reported the school(s) their children attended. We then searched public records for the above information. Test performance data were recoded so that higher scores indicated poorer school-wide performance.

#### Familial disadvantage.

Familial disadvantage was assessed via three indices: maternal reports of total annual household income and maternal and paternal educational attainment. Household income was measured on a 10-point Likert scale (1 = <$10,000 to 10 = >$50,000). Educational attainment was also measured on a 10-point Likert scale (1 = <7 grade to 10 = advanced degree). Scales were recoded so that higher scores indicated greater disadvantage.

### Youth outcomes

#### Psychopathology.

We examined a combination of teacher, maternal, and self-reports of youth psychopathology. The twins’ teacher(s) completed the Achenbach Teacher Report Form (TRF; Achenbach & Rescorla, 2001), one of the most commonly used instruments for assessing youth emotional and behavioral functioning. Teachers rated the extent to which a series of statements described the child’s behavior over the past six months using a three-point scale (0 = never to 2 = often/mostly true). For these analyses, we examined the Rule-Breaking Behavior (RB) scale (e.g., lies; 12 items; α = .70), Aggressive Behavior (AGG) scale (e.g., fights; 20 items; α = .92), and four DSM-oriented scales: oppositional defiant problems (e.g., defiant, stubborn; 6 items; α = .85), conduct Pproblems (e.g., steals; 18 items; α = .86), affective problems (e.g., cries, feels guilty; 14 items; α = .76), and anxiety problems (e.g., fearful; 6 items; α = .73). We also examined the internalizing (INT; 36 items) and externalizing (EXT; 32 items) broadband scales. The teachers of 115 participants were not available for assessment (because the twins were home-schooled or because parental consents to contact the teachers were completed incorrectly, etc.). Our teacher participation rate across both subsamples was 86%, with teacher reports available for 1,551 participants.

The twins’ mothers completed the Child Behavior Checklist, which also assesses the rule breaking (17 items; α = .65), aggressive (18 items; α = .88), oppositional defiant (5 items; α = .76), conduct problems (17 items; α = .82), affective problems (13 items; α = .66), and anxiety problems (6 items; α = .74) scales, as well as the internalizing (32 items) and externalizing (35 items) broadband scales (Achenbach & Rescorla, 2001). Behaviors during the preceding 6 months were rated using the three-point scale described above. Maternal informant-reports were available for 99% of the twins. When maternal and teacher reports were combined, data were available for 2,053 participants (99.7%).

Lastly, the twins reported on their own disordered eating (DE) symptoms via the Minnesota Eating Behavior Survey (MEBS^1^) (von Ranson et al., 2005). The MEBS comprises 30 true/false items corresponding to four symptom dimensions: body dissatisfaction, weight preoccupation, binge eating, and compensatory behavior. We examined the MEBS total score, which demonstrates good psychometric properties in childhood samples (Culbert et al., 2017). In TBED-C, internal consistency (α) was .80.

#### Cognitive.

Twins completed the Test of Word Reading Efficiency (TOWRE), which assesses phonological decoding ability (i.e., mapping sounds to letters) and reading fluency (Torgesen et al., 1999). The TOWRE comprises two subtests: sight word efficiency, which measures the number of real words correctly identified in 45 s, and phonemic decoding efficiency, which measures the number of non-words correctly pronounced in 45 s. Scores were reverse-coded prior to analysis so that higher scores corresponded to poorer performance. The TOWRE has been found to have good psychometric properties (Torgesen et al., 1999) and to predict academic achievement (Paige et al., 2019).

### Data analyses

Using M*plus* 8.4 (Muthén & Muthén, 1998-2019), we first conducted a series of factor analyses to illuminate the underlying relationship among the indicators of disadvantage. Relative fit was evaluated via 3 indices: the Akaike information criterion (AIC; Akaike, 1987), Bayesian information criterion (BIC; Raftery, 1995), and sample-size adjusted Bayesian information criterion (SABIC; Sclove, 1987). For all indices, lower values indicate better model fit. The best-fitting model was indicated by the lowest AIC, BIC, and SABIC values for at least two of the three indices. Absolute model fit was evaluated with the χ^2^ statistic (Bollen, 1989), root mean square error of approximation (RMSEA), standardized root mean squared residual (SRMR), and comparative fit index (CFI). RMSEA values below .06, SRMR values below .08, and CFI values above .95 indicate good model fit (Hu & Bentler, 1999).

We next examined associations between disadvantage and youth outcomes using multilevel modeling (MLM) to account for the nested structure of the data (i.e., twins nested within families). The Mplus Automation Package (Hallquist & Wiley, 2018) in R (R Core Team, 2020) was used to conduct the analyses, with full information maximum likelihood estimation with robust standard errors to account for missing data (Enders & Bandalos, 2001). Prior to analyses, scores on the CBCL and TRF were log-transformed to adjust for positive skew (skew < .9 for all other outcomes). Because MLM coefficients are unstandardized, we also standardized all variables to have a mean of zero and standard deviation of 1.0 prior to analysis to facilitate interpretation of the fixed-effect estimates.

Specification curve analyses (Simonsohn et al., 2020) are an exhaustive modeling strategy that evaluates all reasonable specifications of available data, both to avoid possible bias in investigator decisions and to identify those specifications that are most consequential. We examined the following specifications: (a) measure of disadvantage (income, maternal education, paternal education, ADI, neighbor-reported neighborhood problems, mother-reported neighborhood problems, and subsidized lunch rate), (b) youth outcome (affective problems, anxiety, oppositional defiant problems, conduct problems, RB, AGG, EXT, INT, DE, and both TOWRE subtests), and (c) adjustment for potential demographic covariates (age, sex, ethnicity, or no covariates). (School-wide test performance was not included among the measures of disadvantage due to its high correlation with subsidized lunch rate; see Results for details.)

In total, there were 836 specifications. As recommended by the SCA developers (Simonsohn et al., 2020), we examined median effect sizes, which are more robust to outliers than are means. We also evaluated the portion of *p*-values < .05, as more robust effects would be expected to be significant across a larger proportion of specifications. Three indicators were evaluated to determine statistical significance: 95% confidence intervals for the median effect size, the median *p*-value, and the mean *Z*-score. For the latter, we converted each *p*-value to a *Z*-score and then computed the average *Z*-score (a *Z*-score of 1.96 corresponds to *p* = .05). To accommodate the slightly different sample sizes for various measures, the average *Z*-score was weighted by sample size. Universal evidence of significance across all three indicators (i.e., 95% confidence intervals that did not include zero, a median *p*-value < .05, and an average *Z*-score ≥ 1.96) was interpreted as a statistically significant effect.

## Results

### Descriptive statistics

Descriptive statistics are shown in Table S2 and described in further detail in the Supplement. The at-risk subsample had a significantly lower income ($57,281) relative to the population-based subsample ($72,027; Cohen’s *d* effect size = .38) and the Census mean at the time ($73,373). In the at-risk subsample, 17.1% of families had an advanced degree, whereas 28% did in the population-based subsample. Mean neighborhood poverty rates were 23.4% in the at-risk subsample and 11.4% in the population-based subsample.

### Factor analyses

Correlations were significantly greater than zero among all indicators of disadvantage (see Table 1). Specifically, ADI, subsidized lunch rate, test scores, and neighborhood problems (regardless of informant) were moderately-to-highly correlated with one another, but only moderately correlated with income and parental educational attainment. Given the overlap between subsidized lunch rate and test score average (*r* = .83), we elected to only consider one. Based on theoretical considerations and prior research (e.g., Caldas & Bankston, 1997), we examined subsidized lunch rate in subsequent analyses.

To illuminate the underlying structure of the disadvantage data, we ran a series of exploratory factor analyses (EFAs), specifying between one and three factors. As shown in Table 2, fit improved as the number of factors increased. However, the fit of the two-factor solution was adequate. Given this pattern of results, and our inclusion of only seven indices of disadvantage, we ran a confirmatory factor analysis (CFA) comparing the respective fit of a two-factor and one-factor solution (see Table 2). The two-factor solution was superior to the one-factor solution by comparative fit indices, and the absolute fit indices of the two-factor model were good. The χ^2^ statistic was significant in all models, but given our sample size, its significance does not necessarily indicate substantial misfit (Bentler & Bonett, 1980).

**Figure 1.**
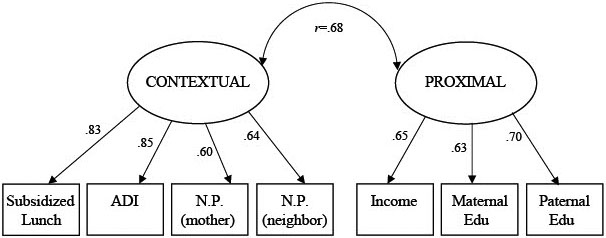
Two-factor confirmatory model of the structure of disadvantage. *Note*. N.P. denotes neighborhood problems. Residual variances are omitted for ease of presentation. No correlations between residual variances were modeled.

Results from the two-factor solution are shown in Figure 1. Subsidized lunch rate, ADI percentile, and mother and neighbor-reported neighborhood problems loaded onto one factor, dubbed contextual disadvantage. Household income and maternal and paternal education loaded onto the other factor, dubbed proximal disadvantage. Put differently, the various indices of disadvantage appear to map onto two broad factors, one representing disadvantage at the contextual-level (i.e., neighborhoods and schools) and one representing disadvantage at the family-level. The two factors were correlated .68 (*p* < .001). Note that this is higher than the observed correlations in Table 1 and reflects the fact that latent factors are necessarily error-free.

### Specification curve analyses

Based on these results, MLM analyses were performed to quantify associations between proximal and contextual disadvantage, respectively, and youth outcomes. Results are shown in Table 3. Proximal disadvantage (operationalized collectively via household income and maternal and paternal education) exhibited a significant association with youth outcomes overall (the median *p*-value was < .001, the average *Z*-score was 4.37, and the 95% confidence intervals did not include zero). This pattern of results persisted to all specific outcomes save Anxiety Problems, which was not significantly associated with proximal disadvantage. Put differently, disadvantage within the home predicted higher levels of nearly all forms of psychopathology and lower scores on both reading subtests. Median effect sizes ranged from .06 to .18, of which 65%–100% were significant at *p* < .05. Likewise, the median *p*-values were < .05 and the average *Z*-scores ranged from 2.39 to 5.94.

Contextual disadvantage (i.e., mother and neighbor-reported neighborhood problems, ADI, and subsidized lunch rate) also predicted youth outcomes overall (the median *p*-value was < .001, the average *Z*-score was 4.02, and the 95% confidence intervals did not include zero). With the exception of anxiety problems and INT, contextual disadvantage was associated with all youth outcomes evaluated herein. Median *p*-values were <.05, average *Z*-scores ranged from 3.12 to 5.28, and confidence intervals did not include zero. Median effect sizes ranged from .09 to .17, of which 87.96%–100% were significant at *p* < .05.

Lastly, we combined the above analyses into a single regression to determine whether the effects were specific to one type of disadvantage. When entered in the same model, proximal and contextual disadvantage significantly incremented each other for all forms of externalizing, DE, and performance on both reading subtests (see Table 3). Even when controlling for the overlap with proximal disadvantage, contextual disadvantage continued to predict all outcomes save affective problems, anxiety problems, and INT. Median effect sizes ranged from .08 to .12, of which 66%–100% were significant at *p* < .05. Average *Z*-scores ranged from 2.61 to 3.48, median *p*-values were <.05, and confidence intervals did not include zero. Similarly, median effect sizes for proximal disadvantage ranged from .08 to .14 (60%–100% of which were significant at *p* < .05), average *Z*-scores ranged from 2.43 to 4.17, and median *p*-values were <.05. For anxiety problems and INT, the effect of proximal disadvantage was not significant.

Notably, MLM results were consistent regardless of which covariates were included in the model (i.e., age, sex, ethnicity, or no covariates). Moreover, when controlling for proximal disadvantage, contextual disadvantage predicted youth outcomes overall in 65%–70% of model specifications. In turn, when we controlled for contextual disadvantage, proximal disadvantage predicted youth outcomes overall in 74%–77% of specifications. The addition of covariates thus had little impact on our results, as the associations between proximal and contextual disadvantage, respectively, and youth outcomes were consistent across models.

## Discussion

The aims of our study were to illuminate the structure underlying multiple, correlated forms of socioeconomic disadvantage and to illuminate the “active ingredients” of disadvantage associated with important youth outcomes. Underlying the seven indicators of disadvantage were two distinct, yet correlated, factors. One factor, comprising household income and maternal and paternal educational attainment, represented socioeconomic disadvantage within the family. The other factor represented socioeconomic disadvantage in the broader school and neighborhood contexts, with moderate-to-high factor loadings for ADI, neighbor and mother-reported neighborhood problems, and subsidized lunch rate. In particular, the high correlation between subsidized lunch rate and ADI percentile (*r* = .70) suggests that disadvantage at the school and neighborhood levels may overlap considerably as indicators of concentrated contextual poverty. While neighborhood and school disadvantage appeared to represent a single construct, there was a clear distinction between disadvantage in the broader context and disadvantage within the home, with all correlations between contextual and proximal indicators, respectively, <.45. In other words, our results support the conceptualization of disadvantage as a multifaceted construct comprising (at least) two distinct dimensions.

We subsequently examined proximal and contextual disadvantage as joint predictors of multiple outcomes. In findings that were largely consistent with our hypotheses, the two broad forms of disadvantage each contributed uniquely to most outcomes. Specifically, proximal and contextual disadvantage incremented one another as predictors of performance on both TOWRE subtests, DE, and scores on all externalizing measures. Thus, youth exposed to disadvantage within their homes and in the broader neighborhood and school contexts demonstrated less proficiency in reading than did their peers who were only exposed to one form of disadvantage, consistent with prior findings that school-level poverty incremented familial poverty in predicting academic difficulties (Caldas & Bankston, 1997). The present findings suggest that the neighborhood context also plays an important role in academic outcomes.

Likewise, joint prediction was consistently observed for all externalizing outcomes under study. Proximal and contextual disadvantage were each uniquely associated with the development of physical aggression, oppositional defiance, nonaggressive rule breaking, and symptoms of conduct disorder. Our findings are consistent with prior work indicating that neighborhood disadvantage confers increased risk for antisocial behavior, even after accounting for familial poverty (Kupersmidt et al., 1995). Results were similar for DE, consistent with prior studies reporting that familial indices of disadvantage (e.g., Becker et al., 2017) and neighborhood characteristics (Mikhail et al., 2021) both confer risk. Our findings also implicate school-level disadvantage, not examined in prior studies of DE, as an important risk factor. Taken together, youth exposed to multiple, co-occurring forms of disadvantage, both within the home and in the broader community, may be at considerable risk for externalizing problems and DE.

In sharp contrast, only proximal disadvantage significantly predicted symptoms of depression. While consistent with prior research implicating poverty as a risk factor for affective disorders (Tracy et al., 2008), these findings suggest that the ‘active ingredients’ of disadvantage for some forms of internalizing are concentrated within the home (although there was a significant, positive association between contextual disadvantage and affective problems when not controlling for proximal disadvantage; see Table 3). By contrast, neither form of disadvantage uniquely predicted anxiety symptoms, and proximal and contextual disadvantage did not jointly predict any form of internalizing. One possible explanation for these findings is that the unique effects of contextual disadvantage on internalizing may not manifest fully until adolescence, when the prevalence of internalizing disorders increases sharply (Holm-Denoma et al., 2014). Although the prevalence of DE also increases during adolescence, prior work has found risk for DE to manifest earlier in development in disadvantaged neighborhoods (Mikhail et al., 2021). Moreover, to the extent that contextual disadvantage indexes access to food (e.g., food deserts or lack of access to healthy foods; Larson et al., 2009), it may be more closely tied to the development of DE than to depression or anxiety. Regardless, our results suggest that, during middle childhood, proximal disadvantage is the more potent predictor of depression, whereas the development of anxiety during childhood may not be robustly tied to exposure to any form of disadvantage (among those examined here).

These results should be interpreted in light of several limitations. First, participants were predominantly white. Although our sample is representative of the child population of Michigan, future studies should examine whether our findings generalize to youth from more diverse racial/ethnic backgrounds. Next, children in our study were twins. Despite prior findings that twins are comparable to singletons on most traits (Christensen & McGue, 2020), two children of the same age necessarily represent a greater financial responsibility than one child does. Indeed, families in our sample are somewhat larger than the average American family, which had 1.93 children at the 2020 Census (http://www.census.gov/). As a result, twin families may be under greater financial strain than families of comparable means but fewer children. Future studies should seek to replicate our results in singleton samples. Nevertheless, this consideration is most relevant to our results for proximal disadvantage (and particularly household income), as we would not expect neighborhoodwide or school-wide disadvantage to be experienced differently by twin families compared to non-twin families. Because the associations between proximal disadvantage and youth outcomes were robust across model specifications and persisted even when controlling for contextual disadvantage, we expect that our results would generalize to non-twin children as well.

Next, we examined data collected at one time point in middle childhood, meaning our results may not generalize to other age groups. This is particularly relevant in light of the substantial increase in internalizing problems and DE during adolescence, particularly among girls (e.g., Holm-Denoma et al., 2014). Externalizing problems, by contrast, tend to decrease throughout childhood and adolescence, at least in recent years (e.g., Carroll et al., 2021). It is unclear whether the various forms of disadvantage increment one another as predictors of the magnitude of this decline. Future work should examine the respective roles of proximal and contextual disadvantage as predictors of behavioral trajectories.

Next, participating mothers reported on household income, their own educational attainment, and youth psychopathology, raising the possibility that shared-informant effects amplified associations with proximal disadvantage. That said, maternal reports of neighborhood problems were examined as one measure of contextual disadvantage as well. In addition, proximal disadvantage significantly predicted all three outcomes that were not based on maternal reports (i.e., child-reported DE and performance on both TOWRE subtests). Given our variety of measures of disadvantage and child outcomes, we believe that our results are unlikely to be due to informant effects. However, subsequent studies should seek to replicate the pattern of joint prediction for externalizing identified here using self-reports.

Lastly, given the high correlation between subsidized lunch rate and average test scores, only one measure of school disadvantage was retained. Its moderate-to-high correlation with all neighborhood measures indicated that subsidized lunch rate overlapped with neighborhood indices as a measure of concentrated poverty. Future work is needed to examine additional indices of school-level disadvantage and to clarify whether any of these indices represent a construct distinct from neighborhood disadvantage.

Despite these limitations, the present study advances our understanding of disadvantage as a multifaceted predictor of psychopathology and cognitive performance during childhood. Our study yielded two important conclusions. First, disadvantage is not a unitary construct but rather comprises at least two distinct dimensions. Studies that seek to illuminate the effects of socioeconomic disadvantage on development should adopt a comprehensive approach by examining disadvantage both within the home and within the broader neighborhood and school contexts. In other words, examining a single index of disadvantage (e.g., household income) will not yield a complete picture of the detrimental effects of exposure to disadvantage in general.

Second, associations with proximal and contextual disadvantage were stronger and more consistent for externalizing outcomes relative to internalizing outcomes. These findings support the distinction between internalizing and externalizing as broad dimensions of psychopathology that evidence disparate etiologies, consistent with prior studies in youth samples (e.g., Martel et al., 2017; Waldman et al., 2016). In addition, prior research has found chronic stress, measured across multiple domains including family and neighborhood, to predict externalizing symptoms during early adolescence, even after accounting for comorbidity with internalizing via the common psychopathology “*p*” factor (Snyder et al., 2019). Taken together, these findings underscore the salience of multiple, co-occurring forms of adversity to the development of externalizing pathology in particular. To the extent that exposure to multiple forms of disadvantage is more closely tied to externalizing than internalizing, we may expect patterns of comorbidity to differ across contexts. Future studies should seek to illuminate how the emergence of externalizing and internalizing pathology, respectively, differs across contexts, as well as when in development the effects of each form of disadvantage are most pronounced.

## Supplementary Material

Supplement

Tables (supplement)

## Figures and Tables

**Table 1.** Correlations among the indicators of disadvantage.

	2.	3.	4.	5.	6.	7.	8.
1. ADI	.50*	.49*	.70*	.61*	.40*	.35*	.42*
2. Neighborhood Problems (neighbor report)	-	.45*	.56*	.53*	.32*	.15*	.20*
3. Neighborhood Problems (mother report)		-	.44*	.41*	.36*	.22*	.20*
4. Subsidized lunch rate			-	.83*	.32*	.27*	.36*
5. Test score average+				-	.26*	.21*	.32*
6. Household income+					-	.43*	.35*
7. Maternal education+						-	.45*
8. Paternal education+							-

*Note*. Bold font and an asterisk indicate *p*<.05. To facilitate the subsequent specification curve analyses, all data were coded such that higher scores represent higher levels of disadvantage (to accomplish this, those indicated by a + were reverse-scored).

**Table 2.** Fit indices for factor analyses.

		Exploratory factor analyses					
	*χ*^*2*^ (*df*)	AIC	BIC	SABIC	-	-	-
1-factor	477.05† (14)	31806.55	31924.77	31858.05			
2-factor	74.59† (8)	31416.09	31568.09	31482.31			
3-factor	12.56† (3)	31364.05	31544.20	31442.53			
		Confirmatory factor analyses					
	*χ*^*2*^ (*df*)	AIC	BIC	SABIC	RMSEA (90% CI)	SRMR	CFI
1-factor	221.05† (14)	31806.55	31924.77	31858.05	.085 (.075-.095)	.068	.871
***2-factor***	76.61† (13)	***31495.76***	***31619.61***	***31549.71***	***.049 (.039-.060)***	***.040***	***.960***

*Note*. † ***χ***^***2***^ is significant at *p* < .05. Bold and italicized font indicates the best-fitting confirmatory model. The 2-factor model comprised one factor representing household income, maternal education, and paternal education, and another representing Area Deprivation Index percentile, neighbor-reported neighborhood problems, mother-reported neighborhood problems, and subsidized lunch rate.

**Table 3.** SCA results: associations between disadvantage and youth outcomes.

		Proximal disadvantage only	Contextual disadvantage only	Proximal disadvantage (controlling for contextual)	Contextual disadvantage (controlling for proximal)
Affective Problems					
	Median ES	**.09** *	**.09** *	**.08** *	.06
	95% CIs	**(.04, .15)**	**(.03, .14)**	**(.01, .14)**	(−.003, .12)
	Median *p* value	**.002**	**.003**	**.016**	.06
	% *p*<.05	**100.00**	**87.96**	**94.66**	42.44
	Avg Z-score	**4.00**	**3.12**	**3.08**	2.00
Anxiety					
	Median ES	.04	.04	.04	.03
	95% CIs	(−.01, .10)	(−.01, .09)	(−.03, .10)	(−.03, .09)
	Median *p* value	.111	.15	.25	.30
	% *p*<.05	8.79	28.71	2.12	21.78
	Avg Z-score	1.28	1.82	.88	1.45
Oppositional Defiant Problems					
	Median ES	**.13** *	**.11** *	**.10** *	**.09** *
	95% CIs	**(.07, .18)**	**(.05, .17)**	**(.04, .16)**	**(.03, .15)**
	Median *p* value	**<.001**	**.001**	**.002**	**.006**
	% *p*<.05	**100.00**	**100.00**	**56.95**	**74.81**
	Avg Z-score	**3.72**	**3.99**	**2.43**	**2.86**
Conduct Problems					
	Median ES	**.17** *	**.16** *	**.14** *	**.11** *
	95% CIs	**(.12, .23)**	**(.11, .22)**	**(.07, .20)**	**(.05, .18)**
	Median *p* value	**<.001**	**<.001**	**<.001**	**<.001**
	% *p*<.05	**100.00**	**100.00**	**100.00**	**100.00**
	Avg Z-score	**5.79**	**5.22**	**4.03**	**3.47**
AGG					
	Median ES	**.14** *	**.13** *	**.10** *	**.09** *
	95% CIs	**(.08, .19)**	**(.07, .18)**	**(.03, .16)**	**(.03, .16)**
	Median *p* value	**<.001**	**<.001**	**.004**	**.005**
	% *p*<.05	**100.00**	**100.00**	**73.37**	**85.65**
	Avg Z-score	**4.25**	**4.50**	**2.82**	**3.12**
RB					
	Median ES	**.18** *	**.17** *	**.13** *	**.12** *
	95% CIs	**(.12, .23)**	**(.10, .23)**	**(.07, .20)**	**(.05, .19)**
	Median *p* value	**<.001**	**<.001**	**<.001**	**<.001**
	% *p*<.05	**100.00**	**100.00**	**100.00**	**100.00**
	Avg Z-score	**5.94**	**5.28**	**4.09**	**3.48**
EXT					
	Median ES	**.16** *	**.14** *	**.12** *	**.10** *
	95% CIs	**(.10, .21)**	**(.09, .20)**	**(.05, .18)**	**(.04, .17)**
	Median *p* value	**<.001**	**<.001**	**.001**	**.002**
	% *p*<.05	**100.00**	**100.00**	**75.49**	**87.43**
	Avg Z-score	**4.98**	**4.92**	**3.32**	**3.36**
INT					
	Median ES	**.06** *	.06	.05	.04
	95% CIs	**(.01, .12)**	(−.001, .11)	(−.01, .12)	(−.02, .11)
	Median *p* value	**.027**	.06	.10	.18
	% *p*<.05	**64.83**	50.98	37.03	24.38
	Avg Z-score	**2.39**	2.27	1.72	1.62
Disordered Eating					
	Median ES	**.12** *	**.12** *	**.09** *	**.08** *
	95% CIs	**(.07, .17)**	**(.07, .17)**	**(.02, .15)**	**(.02, .14)**
	Median *p* value	**<.001**	**<.001**	**.007**	**.01**
	% *p*<.05	**100.00**	**94.68**	**91.66**	**74.04**
	Avg Z-score	**4.31**	**4.01**	**2.81**	**2.61**
TOWRE Sight Word Efficiency					
	Median ES	**.16** *	**.12** *	**.14** *	**.09** *
	95% CIs	**(.11, .22)**	**(.06, .18)**	**(.07, .20)**	**(.03, .16)**
	Median *p* value	**<.001**	**<.001**	**<.001**	**.003**
	% *p*<.05	**100.00**	**94.74**	**100.00**	**80.34**
	Avg Z-score	**5.87**	**4.75**	**4.17**	**3.21**
TOWRE Phonemic Decoding Efficiency					
	Median ES	**.15** *	**.11** *	**.12** *	**.08** *
	95% CIs	**(.10, .21)**	**(.06, .17)**	**(.06, .19)**	**(.02, .14)**
	Median *p* value	**<.001**	**<.001**	**<.001**	**.02**
	% *p*<.05	**100.00**	**93.77**	**100.00**	**66.29**
	Avg Z-score	**5.61**	**4.34**	**4.00**	**2.71**

*Note*. Proximal disadvantage comprises household income and maternal and paternal educational attainment, whereas contextual disadvantage comprises maternal and neighbor reports of neighborhood problems, Area Deprivation Index percentile, and subsidized lunch rate. To facilitate comparison and compute overall effect sizes, all measures of disadvantage were coded so that higher scores represented greater disadvantage, and all outcome measures were coded so that higher scores corresponded to poorer outcomes. We report median effect sizes (ES) and median lower and upper 95% confidence intervals across the various specifications, as well as the proportion of specifications with a *p-*value < .05. We also converted each *p*-value to a Z-score and then computed the average Z-score. The ES that were statistically significant across all indices are bolded with an *.

## Footnotes

1 The Minnesota Eating Behavior Survey (MEBS; previously known as the Minnesota Eating Disorder Inventory [M-EDI]) was adapted and reproduced by special permission of Psychological Assessment Resources, 16204 North Florida Avenue, Lutz, Florida 33549, from the Eating Disorder Inventory (collectively, EDI and EDI-2) by Garner, Olmstead, Polivy, Copyright 1983 by Psychological Assessment Resources. Further reproduction of the MEBS is prohibited without prior permission from Psychological Assessment Resources.
