# Supplementary material for: Identifying the ‘active ingredients’ of socioeconomic disadvantage for youth outcomes in middle childhood
Source: Dev Psychopathol. Author manuscript; Available in PMC 2024 Nov 1. (PMC10915935; doi:10.1017/S0954579423000135)
Supplement: Supplement [file NIHMS1963452-supplement-Supplement.docx]

**Supplement**

**Descriptive Statistics**

Boys scored higher than girls on all psychopathology scales except disordered eating, on which sex differences were not significant. Such findings are consistent with prior results indicating that the female preponderance of internalizing symptoms emerges during puberty (Hayward & Sanborn, 2002). These sex differences were larger for externalizing outcomes (Cohen’s *d* ranged from .27 to .34) than for internalizing (*d*s ranged from .04 to .18). Girls scored higher than boys on the TOWRE Sight Word Efficiency test (*d*=.17, *p*<.05), whereas scores did not differ by sex on TOWRE Phonemic Decoding Efficiency.

Affective problems were more common among older participants (*r*=.08, *p*<.001), whereas conduct problems and disordered eating were somewhat less common (both *r*s were -.05, *p*<.05). Externalizing broadband scores were somewhat lower for older participants (r=-.08, p<.01). Scores on the other scales, including the Internalizing broadband, did not vary significantly by age. Scores on both TOWRE subtests were significantly correlated with age (*r*s were .71 and .63, respectively, both *p*<.001), as expected given that raw scores were used for these analyses.

**Specification Curve Analyses**

Each individual measure of proximal disadvantage was observed to predict youth outcomes overall (i.e., collapsing across all measures of psychopathology and academic performance), with median effect sizes ranging from .10 to .16 (81.66%-93.12% significant at *p* < .05). Median *p-*values were < .05, and average Z-scores ranged from 3.44 to 5.20. Thus, results were robust across specifications of proximal disadvantage. Likewise, results persisted across indices of contextual disadvantage. Each was observed to predict youth outcomes overall, with median effect sizes ranging from .10 to .16 (77.13%-95.58% significant at *p* < .05), mean Z-scores ranging from 2.98 to 4.90, and all median *p-*values < .05.

When controlling for contextual disadvantage, each proximal indicator was also observed to predict youth outcomes overall, with median effect sizes ranging from .08 to .13 (59.21%-88.17% significant), average Z-scores ranging from 2.23 to 3.72, and median *p-*values < .05. Likewise, each contextual indicator predicted youth outcomes overall when controlling for proximal disadvantage, such that median effect sizes were .07 to .14 (60.97%-82.15% significant), average Z-scores were 2.05 to 3.49, and median *p-*values were < .05 (see Table S3). Thus, the associations observed between both broad forms of disadvantage and youth outcomes were robust across specifications of disadvantage.

**Table S1.** Measures included in Area Deprivation Index.

| ***Measure*** |  |
| --- | --- |
|  | 1. Percent of population aged 25 and older with <9 years of education |
|  | 1. Percent of population aged 25 and older with at least a high school diploma |
|  | 1. Percent of population aged 16 and older in white-collar occupations |
|  | 1. Median family income |
|  | 1. Income disparity (ratio of households with <$10,000 income to households with ≥$50,000 income) |
|  | 1. Median home value |
|  | 1. Median gross rent |
|  | 1. Median monthly mortgage |
|  | 1. Percent of housing units owned by occupiers |
|  | 1. Percent of population aged 16 and older who are unemployed |
|  | 1. Percent of families below poverty level |
|  | 1. Percent of population below 150% of the poverty threshold |
|  | 1. Percent of households with children under age 18 headed by a single parent |
|  | 1. Percent of households without a motor vehicle |
|  | 1. Percent of households without a telephone |
|  | 1. Percent of occupied housing units without complete plumbing |
|  | 1. Percent of households with more than 1 person per room |

*Note.* Participating families’ ADI scores were determined by the level of deprivation in their Census block group based on all indices listed above. For additional details, see Singh (2003) and Kind & Buckingham (2018).

**Table S2.** Descriptive statistics for disadvantage and youth outcomes (*N* = 2060 participants).

| **Measure** | ***N*** | **Mean (SD)** | **Range** (possible range) |
| --- | --- | --- | --- |
| ***Disadvantage*** |  |  |  |
| ADI | 2010 | 57.25 (22.68) | 2-99 (1-100) |
| Neighborhood Problems (neighbor report) | 1690 | 25.94 (6.96) | 13-55 (13-65) |
| Neighborhood Problems (mother report) | 1438 | 22.13 (10.44) | 13-65 (13-65) |
| Subsidized lunch rate | 1630 | 44.25 (22.77) | 0-100 (0-100) |
| Test score average | 1406 | 39.52 (23.78) | 0-95 (0-100) |
| Household income | 1958 | - | - |
| Maternal education | 1960 | - | - |
| Paternal education | 1658 | - | - |
| ***Psychopathology*** |  |  |  |
| Affective Problemsǂ | 2055 | 1.11 (1.59) | 0-12 (0-27) |
| Anxiety Problemsǂ | 2055 | 1.19 (1.45) | 0-10 (0-12) |
| Oppositional Defiant Problemsǂ | 2055 | 1.67 (1.71) | 0-10 (0-11) |
| Conduct Problemsǂ | 2055 | 1.44 (2.31) | 0-19.50 (0-35) |
| Aggressionǂ | 2055 | 3.51 (4.09) | 0-32 (0-38) |
| Rule-Breakingǂ | 2055 | 1.22 (1.64) | 0-14 (0-29) |
| Externalizingǂ | 2059 | 4.75 (5.49) | 0-41.50 (0-67) |
| Internalizingǂ | 2059 | 4.68 (4.40) | 0-34.00 (0-68) |
| MEBS Total± | 1981 | 5.49 (4.20) | 0-22 (0-30) |
| ***Cognitive Performance*** |  |  |  |
| TOWRE Sight Word Efficiency | 1991 | 47.53 (22.86) | 0-95 (0-108) |
| TOWRE Phonemic Decoding Efficiency | 1989 | 38.86 (13.86) | 0-61 (0-66) |

ǂMother-teacher combined report. ±Self-report. All measures of disadvantage are coded so that higher scores represent greater disadvantage. Measures of psychopathology and cognitive performance are coded so that higher scores represent poorer outcomes.

**Table S3.** Independent associations of proximal and contextual disadvantage with youth outcomes.

|  | | **Proximal disadvantage, controlling for contextual** | | | **Contextual disadvantage, controlling for proximal** | | | |
| --- | --- | --- | --- | --- | --- | --- | --- | --- |
|  |  | Household income ^a^ | Maternal education ^a^ | Paternal education ^a^ | Subsidized lunch rate ^b^ | ADI ^b^ | Neighbor-reported problems ^a^ | Mother-reported problems ^a^ |
| **Youth outcomes overall** | Median ES | **.13*** | **.08*** | **.11*** | **.08*** | **.10*** | **.07*** | **.14*** |
|  | 95% CIs | **(.06, .19)** | **(.01, .14)** | **(.05, .17)** | **(.01, .15)** | **(.04, .16)** | **(.01, .14)** | **(.07, .22)** |
|  | Median *p*-value | **<.001** | **.017** | **.001** | **.020** | **.001** | **.035** | **<.001** |
|  | % *p* < .05 | **88.17** | **59.21** | **79.47** | **61.52** | **71.40** | **60.97** | **82.15** |
|  | Avg Z-score | **3.72** | **2.23** | **3.15** | **2.26** | **3.08** | **2.05** | **3.49** |

*Note.* We report median effect sizes (ES) and median lower and upper 95% confidence intervals across the various specifications, as well as the proportion of specifications with a *p-*value < .05. We also converted each *p*-value to a Z-score and then computed the average Z-score. ^a^ and ^b^ indicate informant-report and administrative data, respectively. The ES that were statistically significant across all indices are bolded with an *.

**References**

Hayward, C., & Sanborn, K. (2002). Puberty and the emergence of gender differences in psychopathology. *Journal of Adolescent Health*, *30*(4), 49-58.

Kind, A. J., & Buckingham, W. R. (2018). Making neighborhood-disadvantage metrics accessible—the neighborhood atlas. *The New England journal of medicine*, *378*(26), 2456.

Singh, G. K. (2003). Area deprivation and widening inequalities in US mortality, 1969–1998. *American journal of public health*, *93*(7), 1137-1143.
