# Supplementary material for: Identifying the ‘active ingredients’ of socioeconomic disadvantage for youth outcomes in middle childhood
Source: Dev Psychopathol. Author manuscript; Available in PMC 2024 Nov 1. (PMC10915935; doi:10.1017/S0954579423000135)
Supplement: Tables (supplement) [file NIHMS1963452-supplement-Tables__supplement_.docx]

**Supplement**

**Table S1.** Measures included in Area Deprivation Index.

| ***Measure*** |  |
| --- | --- |
|  | 1. Percent of population aged 25 and older with <9 years of education |
|  | 1. Percent of population aged 25 and older with at least a high school diploma |
|  | 1. Percent of population aged 16 and older in white-collar occupations |
|  | 1. Median family income |
|  | 1. Income disparity (ratio of households with <$10,000 income to households with ≥$50,000 income) |
|  | 1. Median home value |
|  | 1. Median gross rent |
|  | 1. Median monthly mortgage |
|  | 1. Percent of housing units owned by occupiers |
|  | 1. Percent of population aged 16 and older who are unemployed |
|  | 1. Percent of families below poverty level |
|  | 1. Percent of population below 150% of the poverty threshold |
|  | 1. Percent of households with children under age 18 headed by a single parent |
|  | 1. Percent of households without a motor vehicle |
|  | 1. Percent of households without a telephone |
|  | 1. Percent of occupied housing units without complete plumbing |
|  | 1. Percent of households with more than 1 person per room |

*Note.* Participating families’ ADI scores were determined by the level of deprivation in their Census block group based on all indices listed above. For additional details, see Singh (2003) and Kind & Buckingham (2018).

**Table S2.** Descriptive statistics for disadvantage and youth outcomes (*N* = 2060 participants).

| **Measure** | ***N*** | **Mean (SD)** | **Range** (possible range) |
| --- | --- | --- | --- |
| ***Disadvantage*** |  |  |  |
| ADI | 2010 | 57.25 (22.68) | 2-99 (1-100) |
| Neighborhood Problems (neighbor report) | 1690 | 25.94 (6.96) | 13-55 (13-65) |
| Neighborhood Problems (mother report) | 1438 | 22.13 (10.44) | 13-65 (13-65) |
| Subsidized lunch rate | 1630 | 44.25 (22.77) | 0-100 (0-100) |
| Test score average | 1406 | 39.52 (23.78) | 0-95 (0-100) |
| Household income | 1958 | - | - |
| Maternal education | 1960 | - | - |
| Paternal education | 1658 | - | - |
| ***Psychopathology*** |  |  |  |
| Affective Problemsǂ | 2055 | 1.11 (1.59) | 0-12 (0-27) |
| Anxiety Problemsǂ | 2055 | 1.19 (1.45) | 0-10 (0-12) |
| Oppositional Defiant Problemsǂ | 2055 | 1.67 (1.71) | 0-10 (0-11) |
| Conduct Problemsǂ | 2055 | 1.44 (2.31) | 0-19.50 (0-35) |
| Aggressionǂ | 2055 | 3.51 (4.09) | 0-32 (0-38) |
| Rule-Breakingǂ | 2055 | 1.22 (1.64) | 0-14 (0-29) |
| Externalizingǂ | 2059 | 4.75 (5.49) | 0-41.50 (0-67) |
| Internalizingǂ | 2059 | 4.68 (4.40) | 0-34.00 (0-68) |
| MEBS Total± | 1981 | 5.49 (4.20) | 0-22 (0-30) |
| ***Cognitive Performance*** |  |  |  |
| TOWRE Sight Word Efficiency | 1991 | 47.53 (22.86) | 0-95 (0-108) |
| TOWRE Phonemic Decoding Efficiency | 1989 | 38.86 (13.86) | 0-61 (0-66) |

ǂMother-teacher combined report. ±Self-report. All measures of disadvantage are coded so that higher scores represent greater disadvantage. Measures of psychopathology and cognitive performance are coded so that higher scores represent poorer outcomes.

**Table S3.** Independent associations of proximal and contextual disadvantage with youth outcomes.

|  | | **Proximal disadvantage, controlling for contextual** | | | **Contextual disadvantage, controlling for proximal** | | | |
| --- | --- | --- | --- | --- | --- | --- | --- | --- |
|  |  | Household income ^a^ | Maternal education ^a^ | Paternal education ^a^ | Subsidized lunch rate ^b^ | ADI ^b^ | Neighbor-reported problems ^a^ | Mother-reported problems ^a^ |
| **Youth outcomes overall** | Median ES | **.13*** | **.08*** | **.11*** | **.08*** | **.10*** | **.07*** | **.14*** |
|  | 95% CIs | **(.06, .19)** | **(.01, .14)** | **(.05, .17)** | **(.01, .15)** | **(.04, .16)** | **(.01, .14)** | **(.07, .22)** |
|  | Median *p*-value | **<.001** | **.017** | **.001** | **.020** | **.001** | **.035** | **<.001** |
|  | % *p* < .05 | **88.17** | **59.21** | **79.47** | **61.52** | **71.40** | **60.97** | **82.15** |
|  | Avg Z-score | **3.72** | **2.23** | **3.15** | **2.26** | **3.08** | **2.05** | **3.49** |

*Note.* We report median effect sizes (ES) and median lower and upper 95% confidence intervals across the various specifications, as well as the proportion of specifications with a *p-*value < .05. We also converted each *p*-value to a Z-score and then computed the average Z-score. ^a^ and ^b^ indicate informant-report and administrative data, respectively. The ES that were statistically significant across all indices are bolded with an *.
